# Crystal structure and hydrogen-bonding patterns in 5-fluoro­cytosinium picrate

**DOI:** 10.1107/S205698901700216X

**Published:** 2017-02-14

**Authors:** Marimuthu Mohana, Packianathan Thomas Muthiah, Colin D. McMillen

**Affiliations:** a School of Chemistry, Bharathidasan University, Tiruchirappalli 620 024, Tamil Nadu, India; b Department of Chemistry, Clemson University, 379 H.L. Hunter Laboratories, Clemson, SC 29634, USA

**Keywords:** crystal structure, 5-fluoro­cytosine, picrate, hydrogen bonding, bifurcated inter­actions

## Abstract

In the crystal structure of the title compound, 5-fluoro­cytosinium picrate, C_4_H_5_FN_3_O^+^·C_6_H_2_N_3_O_7_^−^, one N heteroatom of the 5-fluoro­cytosine (5FC) ring is protonated. The 5FC ring forms a dihedral angle of 19.97 (11)° with the ring of the picrate (PA^−^) anion. In the crystal, the 5FC^+^ cation inter­acts with the PA^−^ anion through three-centre N—H⋯O hydrogen bonds, forming two conjoined rings having *R*_2_^1^(6) and *R*_1_^2^(6) motifs, and is extended by N—H⋯O hydrogen bonds and C—H⋯O inter­actions into a two-dimensional sheet structure lying parallel to (001). Also present in the crystal structure are weak C—F⋯π inter­actions.

## Abstract 2

In the crystal, the 5FC^+^ cation inter­acts with the PA^−^ anion through three-centre N—H⋯O hydrogen bonds, forming two conjoined rings having 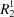
(6) and 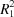
(6) motifs, and is extended by N—H⋯O hydrogen bonds and C—H⋯O inter­actions into a two-dimensional sheet structure lying parallel to (001). Also present in the crystal structure are weak C—F⋯π inter­actions.

## Chemical context   

Crystal engineering is defined as the rational design of crystalline solids through control of inter­molecular inter­actions (hydrogen bonding, hydro­phobic forces, van der Waals forces, π–π inter­actions and electrostatic forces). New solid forms of pharmaceuticals are designed using the crystal engineering approach. These engineered solids have technological and legal importance. Among the inter­molecular inter­actions, hydrogen bonding is the master key for mol­ecular recognition in biological systems because of its strength and directionality (Almarsson & Zaworoko, 2004 ▸; Desiraju, 1995 ▸). It plays a dominant role in mol­ecular aggregates (Samuel, 1997 ▸; Tutughamiarso & Egert, 2012 ▸) and three-dimensional structure, stability and function of biomacromolecules (Gould, 1986 ▸). In particular, pyrimidine derivatives are used in the treatment of anti­viral, anti­fungal, anti­tumor and cardiovascular diseases. 5-fluoro­cytosine (5FC) is a synthetic anti­mycotic compound, first synthesized in 1957 and widely used as an anti­tumor agent it is also active against fungal infection (Heidelberger *et al.*, 1957 ▸; Portalone & Colapietro, 2007 ▸; Vermes *et al.*, 2000 ▸). It becomes active by deamination of 5FC into 5-fluoro­uracil by the enzyme cytosine deaminase (CD) and inhibits RNA and DNA synthesis (Morschhäuser, 2003 ▸). Picric acid forms charge-transfer complexes with many organic compounds. It functions not only as an acceptor to form π-stacking complexes with aromatic biomolecules, but also as an acidic ligand to form salts with polar biomolecules through specific electrostatic hydrogen-bonding inter­actions (In *et al.*, 1997 ▸). The present work is focused on the understanding of supra­molecular hydrogen-bonding patterns exhibited by the inter­action of 5FC and picric acid, giving the (1:1) title salt, C_4_H_5_FN_3_O^+^·C_6_H_2_N_3_O_7_^−^ whose structure and hydrogen-bonding patterns are reported on herein.
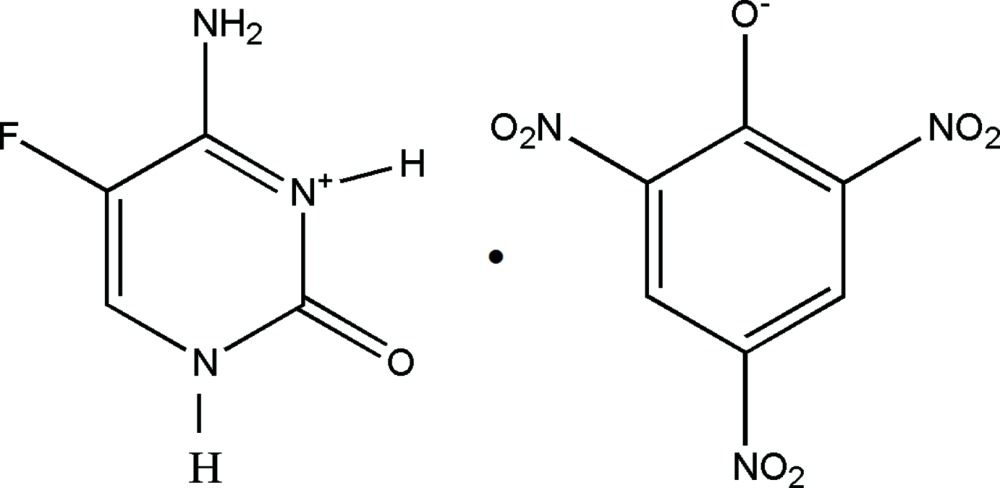


## Structural commentary   

**Figure 1:**
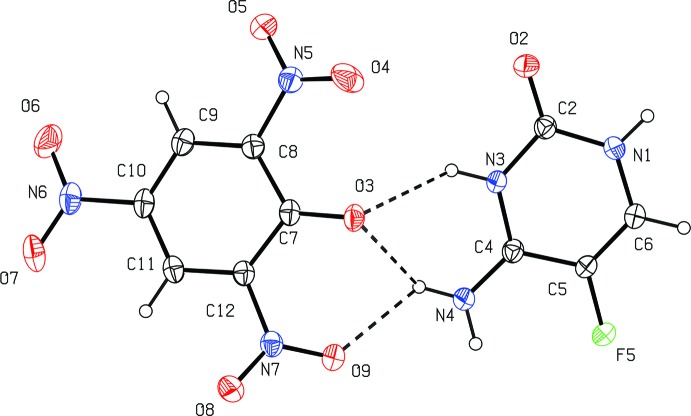
The naming scheme for the 5FC^+^ cation and the PA^−^ anion in the title compound, showing 30% probability level displacement ellipsoids. Dashed lines represent hydrogen bonds.

The asymmetric unit contains one 5-fluoro­cytosinium cation (5FC^+^) and one picrate anion (PA^−^) (Fig. 1 ▸). The 5-fluoro­cytosine cation is protonated at the N3 atom, as is evident from the widening of the corresponding inter­nal angle from 120.8 (5)° to 125.37 (17)° compared to neutral 5FC (Louis *et al.*, 1982 ▸). The dihedral angle between the planes of the rigs in the cation and anion is 19.97 (11)°. In the picrate (PA^−^) anion, the nitro groups lie variously out of the parent benzene ring, with torsion angles C9—C8—N5—O4, C9—C10—N6—O7 and C11—C12—N7—O9 of 166.2 (2), −171.7 (2) and 147.2 (2)°, respectively.

## Supra­molecular features   

In this crystal structure, the N4-amino group and protonated N3 atom of the 5FC^+^ cation inter­act with atoms O3 and O9 of the picrate anion through three-centre N—H⋯O hydrogen bonds, forming two fused-ring motifs with graph-sets 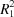
(6) and 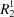
(6) (Fig. 1 ▸). One of the N4-amino hydrogen atoms of the 5FC^+^ cation acts as a three-centre donor and the O3 atom of the PA^−^ anion acts as a three-centre acceptor. This type of inter­action has also been reported in the crystal structures of 2-amino-4,6-di­methyl­pyrimidinium picrate (Subashini *et al.*, 2006 ▸) and 2-amino-4,6- di­meth­oxy­pyrimidinium picrate, pyrimethaminium picrate dimethyl sulfoxide (Thanigaimani *et al.*, 2009 ▸). Similarly, the other hetero nitro­gen atom (N1) of the cation and both the phenolate O3^i^ and a nitro O4^i^ atom of a PA^−^ anion form an 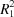
(6) ring motif through N—H⋯O hydrogen bonds with a second C—H⋯O4^i^ inter­action, closing an 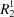
(5) ring (Table 1 ▸). A similar type of inter­action has also been observed in the crystal structure of cytosinium hydrogen chloro­anilate monohydrate (Gotoh *et al.*, 2006 ▸).

**Figure 2:**
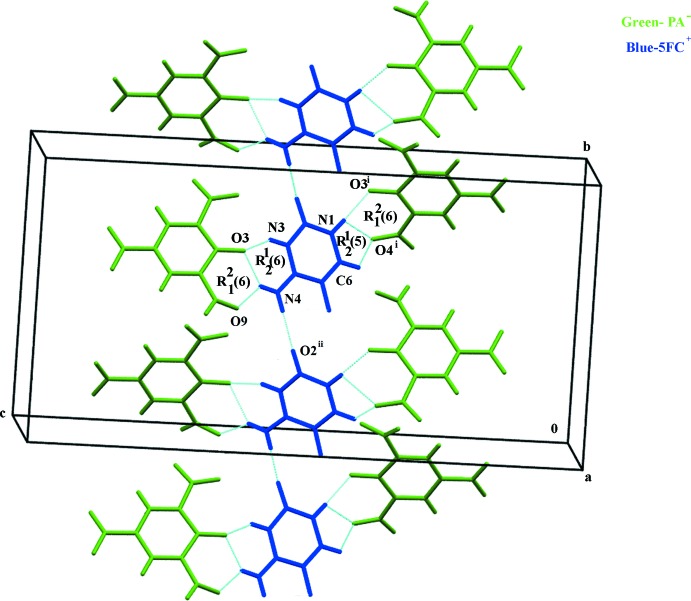
A view of the supra­molecular network formed *via* N—H⋯O and C—H⋯O inter­actions. Dashed lines represent hydrogen bonds. For symmetry codes, see Table 1 ▸.

**Figure 3:**
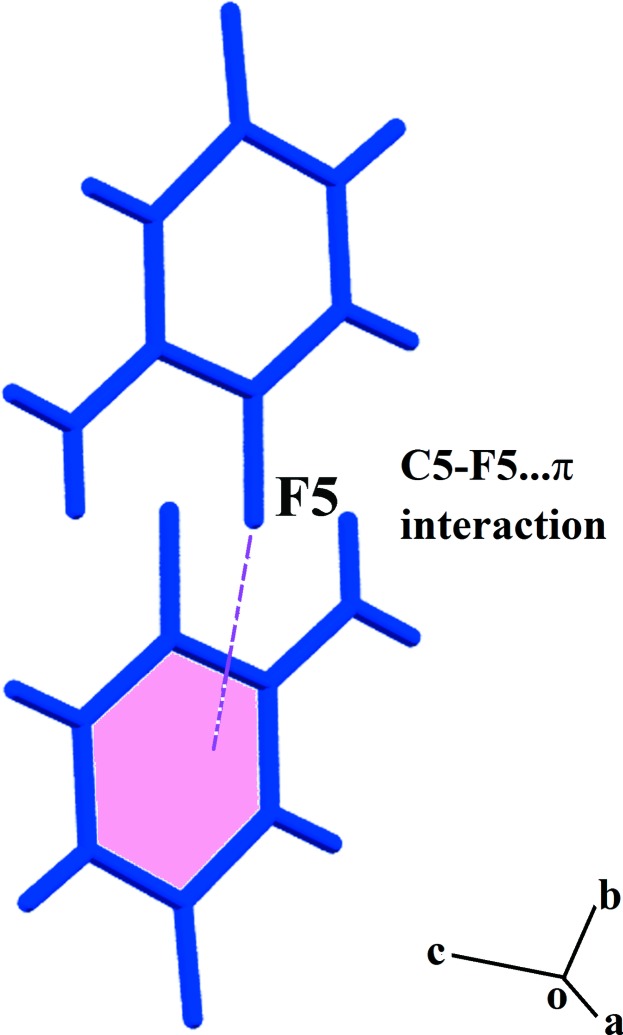
A view of the C5—F5⋯π inter­action between 5FC^+^ cations.

Further, the symmetry-related O2^ii^ atom and the amino group of the 5FC^+^ cation are connected through an N—H⋯O hydrogen bond, forming a two-dimensional supra­molecular network lying parallel to (001) (Fig. 2 ▸). Also present in the crystal structure is a weak C5—F5⋯π inter­action (Fig. 3 ▸) between 5FC^+^ cations [C5⋯*Cg* ^iv^ = 3.4002 (19) Å; C—F⋯*Cg* = 88.34 (12)°, where *Cg* is the centroid of the N1–C6 ring; symmetry code: (iv) −*x*, −*y*, −*z* + 1]. A similar angle [90.5 (2)°] has been reported for a C—F⋯*Cg* inter­action in an acridinium tri­fluoro­methane sulfonate compound (Sikorski *et al.*, 2005 ▸).

## Database survey   

The crystal structures of 5-fluoro­cytosine monohydrates (Louis *et al.*, 1982 ▸; Portalone & Colapietro, 2006 ▸; Portalone & Colapietro, 2007 ▸; Portalone, 2011 ▸), polymorphs (Hulme & Tocher, 2006 ▸; Tutughamiarso & Egert, 2012 ▸), salts (Perumalla *et al.*, 2013 ▸) and co-crystals (Tutughamiarso *et al.*, 2012 ▸; da Silva *et al.*, 2014 ▸) have been reported in the literature. From our laboratory, 5-fluoro­cytosinium salicylate (Prabakaran *et al.*, 2001 ▸), 5-fluoro­cytosinium 3-hy­droxy­picolinate (Karthikeyan *et al.*, 2014 ▸) and 5-fluoro­cytosine melamine (Mohana *et al.*, 2016 ▸) have been reported. Various salts and co-crystals of picric acid have also been reported in the literature (Subashini *et al.*, 2006 ▸; Thanigaimani *et al.*, 2009 ▸; Nagata *et al.*, 1995 ▸; Smith *et al.*, 2004 ▸; Gotoh *et al.*, 2004 ▸).

## Synthesis and crystallization   

A hot aqueous solution of 5-fluoro­cytosine (32 mg) and picric acid (57 mg) were mixed in a 1:1 molar ratio. The resulting solution was warmed to 353 K wrong symmetry description - inversion centre in central benzene ring over a water bath for half an hour and kept for slow evaporation. After a week, colourless prismatic crystals were obtained.

## Refinement   

Crystal data, data collection and structure refinement details are summarized in Table 2 ▸. All hydrogen atoms were positioned geometrically (C—H = 0.95 Å and N—H = 0.88 Å) and were refined using a riding model with *U*_iso_(H) = 1.2*U*_eq_(parent atom).

## Supplementary Material

Crystal structure: contains datablock(s) I. DOI: 10.1107/S205698901700216X/zs2375sup1.cif

Structure factors: contains datablock(s) I. DOI: 10.1107/S205698901700216X/zs2375Isup2.hkl

Supporting information file. DOI: 10.1107/S205698901700216X/zs2375Isup3.cml

CCDC reference: 1531927

Additional supporting information:  crystallographic information; 3D view; checkCIF report

## Figures and Tables

**Table 1:** Hydrogen-bond geometry (Å, °)

*D*—H⋯*A*	*D*—H	H⋯*A*	*D*⋯*A*	*D*—H⋯*A*
N1—H1⋯O3^i^	0.88	1.92	2.794 (3)	175
N1—H1⋯O4^i^	0.88	2.56	3.021 (3)	114
N3—H3⋯O3	0.88	2.22	2.915 (2)	136
N4—H4*A*⋯O3	0.88	2.10	2.828 (2)	139
N4—H4*A*⋯O9	0.88	2.18	2.782 (3)	125
N4—H4*B*⋯O2^ii^	0.88	1.96	2.832 (3)	171
C6—H6⋯O4^i^	0.95	2.51	3.003 (3)	113

Symmetry codes: (i) 
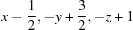
; (ii) 
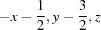
.

**Table 2:** Experimental details

Crystal data	
Chemical formula	C_4_H_5_FN_3_O^+^·C_6_H_2_N_3_O_7_^−^
*M*_r_	358.22
Crystal system, space group	Orthorhombic, *P* *b* *c* *a*
Temperature (K)	200
*a*, *b*, *c* (Å)	7.7463 (15), 13.235 (3), 25.642 (5)
*V* (Å^3^)	2628.9 (9)
*Z*	8
Radiation type	Mo *K*α
μ (mm^−1^)	0.17
Crystal size (mm)	0.65 × 0.58 × 0.20

Data collection	
Diffractometer	Rigaku AFC-8S
Absorption correction	Multi-scan multi-scan
*T*_min_, *T*_max_	0.899, 0.967
No. of measured, independent and observed [*I* > 2σ(*I*)] reflections	21815, 2779, 2367
*R*_int_	0.041
(sin θ/λ)_max_ (Å^−1^)	0.633

Refinement	
*R*[*F* ^2^ > 2σ(*F* ^2^)], *wR*(*F* ^2^), *S*	0.058, 0.174, 1.09
No. of reflections	2779
No. of parameters	226
H-atom treatment	H-atom parameters constrained
Δρ_max_, Δρ_min_ (e Å^−3^)	0.28, −0.36

Computer programs: *CrystalClear* (Rigaku/MSC, 2008 ▸), *SHELXS97* (Sheldrick, 2008 ▸), *SHELXL2016* (Sheldrick, 2015 ▸), *PLATON* (Spek, 2009 ▸), *Mercury* (Macrae *et al.*, 2008 ▸), *POVRay* (Cason, 2004 ▸) and *publCIF* (Westrip, 2010 ▸).

## supplementary crystallographic information

### Crystal data

**Table:**

C_4_H_5_FN_3_O^+^·C_6_H_2_N_3_O_7_^−^	*D*_x_ = 1.810 Mg m^−^^3^
*M**_r_* = 358.22	Mo *K*α radiation, λ = 0.71073 Å
Orthorhombic, *P**b**c**a*	Cell parameters from 2779 reflections
*a* = 7.7463 (15) Å	θ = 3.1–26.7°
*b* = 13.235 (3) Å	µ = 0.17 mm^−^^1^
*c* = 25.642 (5) Å	*T* = 200 K
*V* = 2628.9 (9) Å^3^	Prism, colorless
*Z* = 8	0.65 × 0.58 × 0.20 mm
*F*(000) = 1456	

### Data collection

**Table:**

Rigaku AFC-8S diffractometer	2779 independent reflections
Radiation source: fine focus sealed tube	2367 reflections with *I* > 2σ(*I*)
Detector resolution: 14.6199 pixels mm^-1^	*R*_int_ = 0.041
ω scans	θ_max_ = 26.7°, θ_min_ = 3.1°
Absorption correction: multi-scan multi-scan	*h* = −9→7
*T*_min_ = 0.899, *T*_max_ = 0.967	*k* = −16→16
21815 measured reflections	*l* = −32→32

### Refinement

**Table:**

Refinement on *F*^2^	0 restraints
Least-squares matrix: full	Hydrogen site location: inferred from neighbouring sites
*R*[*F*^2^ > 2σ(*F*^2^)] = 0.058	H-atom parameters constrained
*wR*(*F*^2^) = 0.174	*w* = 1/[σ^2^(*F*_o_^2^) + (0.1025*P*)^2^ + 1.2366*P*] where *P* = (*F*_o_^2^ + 2*F*_c_^2^)/3
*S* = 1.09	(Δ/σ)_max_ < 0.001
2779 reflections	Δρ_max_ = 0.28 e Å^−^^3^
226 parameters	Δρ_min_ = −0.36 e Å^−^^3^

### Special details

**Table:**

Geometry. All esds (except the esd in the dihedral angle between two l.s. planes) are estimated using the full covariance matrix. The cell esds are taken into account individually in the estimation of esds in distances, angles and torsion angles; correlations between esds in cell parameters are only used when they are defined by crystal symmetry. An approximate (isotropic) treatment of cell esds is used for estimating esds involving l.s. planes.

### Fractional atomic coordinates and isotropic or equivalent isotropic displacement parameters (Å^2^)

**Table:**

	*x*	*y*	*z*	*U*_iso_*/*U*_eq_	
O3	0.4311 (2)	0.65661 (11)	0.61738 (6)	0.0381 (4)	
O9	0.5493 (2)	0.46460 (13)	0.62650 (7)	0.0440 (4)	
O7	0.5646 (3)	0.58196 (18)	0.85486 (7)	0.0566 (5)	
O8	0.7432 (2)	0.45799 (14)	0.68664 (7)	0.0469 (4)	
C7	0.4418 (3)	0.65628 (15)	0.66650 (8)	0.0317 (5)	
N7	0.6116 (2)	0.49512 (14)	0.66734 (7)	0.0353 (4)	
C12	0.5282 (3)	0.57726 (16)	0.69555 (8)	0.0317 (4)	
C8	0.3714 (3)	0.73340 (16)	0.70030 (8)	0.0338 (5)	
C10	0.4631 (3)	0.65060 (18)	0.77790 (8)	0.0365 (5)	
O4	0.2970 (3)	0.83845 (18)	0.63172 (8)	0.0664 (7)	
N5	0.2811 (3)	0.81933 (15)	0.67800 (8)	0.0390 (4)	
O5	0.1900 (3)	0.87035 (16)	0.70710 (7)	0.0595 (6)	
C11	0.5410 (3)	0.57451 (16)	0.74889 (8)	0.0344 (5)	
H11	0.601989	0.521474	0.765720	0.041*	
O6	0.3831 (4)	0.70675 (18)	0.85946 (8)	0.0733 (7)	
N6	0.4708 (3)	0.64684 (16)	0.83447 (8)	0.0448 (5)	
C9	0.3783 (3)	0.72956 (17)	0.75414 (9)	0.0366 (5)	
H9	0.325057	0.780799	0.774507	0.044*	
F5	0.2136 (2)	0.44284 (10)	0.45073 (6)	0.0484 (4)	
O2	0.0618 (2)	0.81443 (11)	0.51702 (7)	0.0412 (4)	
N3	0.1841 (2)	0.66094 (13)	0.53164 (6)	0.0320 (4)	
H3	0.209477	0.679151	0.563747	0.038*	
N1	0.0672 (3)	0.69947 (14)	0.45092 (7)	0.0371 (4)	
H1	0.019456	0.742373	0.429023	0.044*	
N4	0.3114 (3)	0.50588 (14)	0.54809 (7)	0.0383 (4)	
H4A	0.338817	0.526201	0.579703	0.046*	
H4B	0.339680	0.444694	0.537710	0.046*	
C2	0.1011 (3)	0.73142 (15)	0.50045 (8)	0.0325 (5)	
C4	0.2293 (3)	0.56620 (15)	0.51674 (8)	0.0316 (4)	
C5	0.1785 (3)	0.53785 (16)	0.46564 (8)	0.0348 (5)	
C6	0.1035 (3)	0.60450 (18)	0.43367 (9)	0.0392 (5)	
H6	0.075454	0.585605	0.398941	0.047*	

### Atomic displacement parameters (Å^2^)

**Table:**

	*U*^11^	*U*^22^	*U*^33^	*U*^12^	*U*^13^	*U*^23^
O3	0.0529 (10)	0.0359 (8)	0.0256 (7)	−0.0065 (7)	−0.0044 (6)	0.0023 (6)
O9	0.0528 (10)	0.0445 (9)	0.0347 (8)	0.0036 (7)	−0.0071 (7)	−0.0055 (7)
O7	0.0523 (11)	0.0861 (15)	0.0315 (9)	−0.0037 (10)	−0.0075 (8)	0.0120 (9)
O8	0.0448 (9)	0.0477 (10)	0.0483 (10)	0.0065 (7)	−0.0101 (8)	0.0000 (8)
C7	0.0347 (10)	0.0337 (10)	0.0268 (10)	−0.0097 (8)	−0.0011 (8)	0.0030 (7)
N7	0.0386 (10)	0.0369 (9)	0.0305 (9)	−0.0042 (7)	−0.0001 (7)	0.0035 (7)
C12	0.0335 (10)	0.0331 (10)	0.0286 (10)	−0.0057 (8)	−0.0010 (8)	0.0025 (8)
C8	0.0373 (11)	0.0315 (10)	0.0327 (10)	−0.0057 (8)	−0.0022 (8)	0.0014 (8)
C10	0.0408 (12)	0.0434 (12)	0.0252 (10)	−0.0119 (9)	−0.0015 (8)	0.0014 (8)
O4	0.0806 (15)	0.0708 (14)	0.0478 (11)	0.0296 (11)	0.0199 (10)	0.0262 (10)
N5	0.0424 (10)	0.0379 (10)	0.0368 (10)	−0.0034 (8)	0.0003 (8)	0.0009 (8)
O5	0.0814 (15)	0.0547 (12)	0.0424 (10)	0.0228 (10)	−0.0041 (9)	−0.0079 (8)
C11	0.0357 (11)	0.0380 (10)	0.0296 (10)	−0.0076 (8)	−0.0047 (8)	0.0058 (8)
O6	0.122 (2)	0.0654 (13)	0.0320 (9)	0.0087 (13)	0.0120 (11)	−0.0020 (9)
N6	0.0538 (12)	0.0518 (12)	0.0287 (10)	−0.0135 (10)	−0.0011 (9)	0.0027 (8)
C9	0.0396 (12)	0.0386 (11)	0.0317 (11)	−0.0097 (9)	0.0015 (8)	−0.0019 (8)
F5	0.0673 (10)	0.0373 (7)	0.0404 (8)	0.0123 (6)	−0.0140 (7)	−0.0128 (6)
O2	0.0538 (10)	0.0292 (8)	0.0406 (9)	0.0029 (7)	−0.0023 (7)	−0.0004 (6)
N3	0.0404 (10)	0.0302 (9)	0.0252 (8)	0.0019 (7)	−0.0033 (7)	−0.0028 (6)
N1	0.0491 (11)	0.0347 (9)	0.0274 (9)	0.0077 (8)	−0.0028 (8)	0.0032 (7)
N4	0.0514 (11)	0.0310 (9)	0.0324 (9)	0.0053 (8)	−0.0094 (8)	−0.0031 (7)
C2	0.0380 (11)	0.0308 (11)	0.0288 (10)	−0.0023 (8)	0.0005 (8)	0.0012 (8)
C4	0.0360 (10)	0.0303 (10)	0.0285 (10)	−0.0039 (8)	−0.0006 (8)	0.0000 (8)
C5	0.0439 (11)	0.0312 (10)	0.0294 (10)	0.0031 (8)	−0.0032 (9)	−0.0057 (8)
C6	0.0473 (12)	0.0418 (12)	0.0284 (10)	0.0055 (10)	−0.0053 (9)	−0.0049 (8)

### Geometric parameters (Å, º)

**Table:**

O3—C7	1.262 (3)	O6—N6	1.225 (3)
O9—N7	1.222 (3)	C9—H9	0.9500
O7—N6	1.240 (3)	F5—C5	1.342 (2)
O8—N7	1.235 (3)	O2—C2	1.217 (3)
C7—C8	1.446 (3)	N3—C4	1.357 (3)
C7—C12	1.448 (3)	N3—C2	1.387 (3)
N7—C12	1.457 (3)	N3—H3	0.8800
C12—C11	1.372 (3)	N1—C6	1.362 (3)
C8—C9	1.383 (3)	N1—C2	1.364 (3)
C8—N5	1.452 (3)	N1—H1	0.8800
C10—C9	1.377 (3)	N4—C4	1.299 (3)
C10—C11	1.389 (3)	N4—H4A	0.8800
C10—N6	1.453 (3)	N4—H4B	0.8800
O4—N5	1.219 (3)	C4—C5	1.419 (3)
N5—O5	1.229 (3)	C5—C6	1.337 (3)
C11—H11	0.9500	C6—H6	0.9500
			
O3—C7—C8	124.81 (19)	C10—C9—C8	119.2 (2)
O3—C7—C12	123.1 (2)	C10—C9—H9	120.4
C8—C7—C12	112.08 (18)	C8—C9—H9	120.4
O9—N7—O8	122.5 (2)	C4—N3—C2	125.37 (17)
O9—N7—C12	119.83 (18)	C4—N3—H3	117.3
O8—N7—C12	117.63 (18)	C2—N3—H3	117.3
C11—C12—C7	124.4 (2)	C6—N1—C2	123.29 (18)
C11—C12—N7	116.32 (18)	C6—N1—H1	118.4
C7—C12—N7	119.24 (18)	C2—N1—H1	118.4
C9—C8—C7	123.9 (2)	C4—N4—H4A	120.0
C9—C8—N5	116.14 (19)	C4—N4—H4B	120.0
C7—C8—N5	119.89 (18)	H4A—N4—H4B	120.0
C9—C10—C11	121.4 (2)	O2—C2—N1	123.8 (2)
C9—C10—N6	119.2 (2)	O2—C2—N3	121.46 (19)
C11—C10—N6	119.5 (2)	N1—C2—N3	114.70 (18)
O4—N5—O5	122.3 (2)	N4—C4—N3	121.33 (19)
O4—N5—C8	119.8 (2)	N4—C4—C5	123.0 (2)
O5—N5—C8	117.87 (19)	N3—C4—C5	115.65 (19)
C12—C11—C10	118.9 (2)	C6—C5—F5	122.10 (19)
C12—C11—H11	120.6	C6—C5—C4	120.8 (2)
C10—C11—H11	120.6	F5—C5—C4	117.05 (19)
O6—N6—O7	123.5 (2)	C5—C6—N1	120.0 (2)
O6—N6—C10	118.5 (2)	C5—C6—H6	120.0
O7—N6—C10	117.9 (2)	N1—C6—H6	120.0
			
O4—N5—C8—C7	−16.2 (3)	O3—C7—C8—C9	177.2 (2)
O4—N5—C8—C9	166.2 (2)	C12—C7—C8—N5	179.5 (2)
O5—N5—C8—C7	163.6 (2)	C12—C7—C8—C9	−3.0 (3)
O5—N5—C8—C9	−14.0 (3)	O3—C7—C12—N7	1.8 (3)
O6—N6—C10—C9	9.2 (4)	O3—C7—C12—C11	−179.6 (2)
O6—N6—C10—C11	−170.7 (2)	C8—C7—C12—N7	−178.00 (19)
O7—N6—C10—C9	−171.7 (2)	N5—C8—C9—C10	−179.5 (2)
O7—N6—C10—C11	8.4 (3)	C7—C8—C9—C10	3.0 (4)
O8—N7—C12—C7	147.0 (2)	C8—C9—C10—N6	179.8 (2)
O8—N7—C12—C11	−31.8 (3)	C8—C9—C10—C11	−0.3 (4)
O9—N7—C12—C7	−34.0 (3)	N6—C10—C11—C12	178.0 (2)
O9—N7—C12—C11	147.2 (2)	C9—C10—C11—C12	−1.9 (3)
C2—N1—C6—C5	−1.1 (4)	C10—C11—C12—N7	−179.6 (2)
C6—N1—C2—O2	−176.4 (2)	C10—C11—C12—C7	1.7 (4)
C6—N1—C2—N3	3.1 (3)	N3—C4—C5—F5	−176.70 (18)
C2—N3—C4—C5	−3.0 (3)	N3—C4—C5—C6	5.1 (3)
C4—N3—C2—O2	178.6 (2)	N4—C4—C5—F5	2.2 (3)
C4—N3—C2—N1	−0.9 (3)	N4—C4—C5—C6	−175.9 (2)
C2—N3—C4—N4	178.0 (2)	F5—C5—C6—N1	178.7 (2)
O3—C7—C8—N5	−0.2 (3)	C4—C5—C6—N1	−3.3 (4)
C8—C7—C12—C11	0.7 (3)		

### Hydrogen-bond geometry (Å, º)

**Table:**

*D*—H···*A*	*D*—H	H···*A*	*D*···*A*	*D*—H···*A*
N1—H1···O3^i^	0.88	1.92	2.794 (3)	175
N1—H1···O4^i^	0.88	2.56	3.021 (3)	114
N3—H3···O3	0.88	2.22	2.915 (2)	136
N4—H4*A*···O3	0.88	2.10	2.828 (2)	139
N4—H4*A*···O9	0.88	2.18	2.782 (3)	125
N4—H4*B*···O2^ii^	0.88	1.96	2.832 (3)	171
C6—H6···O4^i^	0.95	2.51	3.003 (3)	113

Symmetry codes: (i) *x*−1/2, −*y*+3/2, −*z*+1; (ii) −*x*−1/2, *y*−3/2, *z*.
